# A new computational strategy for predicting essential genes

**DOI:** 10.1186/1471-2164-14-910

**Published:** 2013-12-21

**Authors:** Jian Cheng, Wenwu Wu, Yinwen Zhang, Xiangchen Li, Xiaoqian Jiang, Gehong Wei, Shiheng Tao

**Affiliations:** 1 College of Life Science, State Key Laboratory of Crop Stress Biology for Arid Areas, Northwest A&F University, Yangling, Shaanxi, China; 2 Bioinformatics Center, Northwest A&F University, Yangling 712100, Shaanxi, China; 3 Key Laboratory of Food Safety Research, Institute for Nutritional Sciences, Shanghai Institutes for Biological Sciences, Chinese Academy of Sciences, University of Chinese Academy of Sciences, Shanghai 200031, China; 4 College of Life Science, Northwest A&F University, Yangling 712100, Shaanxi, China; 5 College of Science, Northwest A&F University, Yangling 712100, Shaanxi, China

**Keywords:** Essential genes, Naïve Bayes, Support vector machine, Gene essentiality

## Abstract

**Background:**

Determination of the minimum gene set for cellular life is one of the central goals in biology. Genome-wide essential gene identification has progressed rapidly in certain bacterial species; however, it remains difficult to achieve in most eukaryotic species. Several computational models have recently been developed to integrate gene features and used as alternatives to transfer gene essentiality annotations between organisms.

**Results:**

We first collected features that were widely used by previous predictive models and assessed the relationships between gene features and gene essentiality using a stepwise regression model. We found two issues that could significantly reduce model accuracy: (i) the effect of multicollinearity among gene features and (ii) the diverse and even contrasting correlations between gene features and gene essentiality existing within and among different species. To address these issues, we developed a novel model called feature-based weighted Naïve Bayes model (FWM), which is based on Naïve Bayes classifiers, logistic regression, and genetic algorithm. The proposed model assesses features and filters out the effects of multicollinearity and diversity. The performance of FWM was compared with other popular models, such as support vector machine, Naïve Bayes model, and logistic regression model, by applying FWM to reciprocally predict essential genes among and within 21 species. Our results showed that FWM significantly improves the accuracy and robustness of essential gene prediction.

**Conclusions:**

FWM can remarkably improve the accuracy of essential gene prediction and may be used as an alternative method for other classification work. This method can contribute substantially to the knowledge of the minimum gene sets required for living organisms and the discovery of new drug targets.

## Background

Essential genes, as a minimal gene subset in organisms, are required for survival, development, or fertility [1,2]. Therefore, the prediction and identification of such genes is not only interesting but also of theoretical and practical significance. Enhanced knowledge of essential genes promotes an understanding of the primary structure of the complex gene regulatory network in a cell [3-5] and helps elucidate the relationship between genotype and phenotype [6,7], identify human diseases [8], discover potential drug targets in novel pathogens [9,10], and re-engineer microorganisms [11,12].

Two types of approaches are mainly used to predict and identify essential genes: experimental laboratory techniques and computational techniques. The former is randomly or systematically used to inactivate potential essential genes, and gene essentiality could be determined based on the living situation of the organism. General gene disruption strategies include single gene knockouts [13], conditional knockouts [14], RNA interference [15], and transposon mutagenesis [16]. Unfortunately, experimental techniques have significant drawbacks, such as long durations and high costs. In addition, the spectrum of gene essentiality varies under different growth conditions [6,17].

Computational techniques have become popular over the past years for several reasons. First, known essential genes from dozens of microorganisms provide instructional and training materials. Second, the available genome sequences obtained by high-throughput sequencing provide unprecedented opportunities for investigating the minimal subset of genes in various organisms. Finally and most importantly, the development of bioinformatics tools improves our capability for exploring essential genes.

Several prediction models have been developed in silico to identify essential genes. Among these models, the simplest one is prediction of essential genes based on the known essentiality of homologous genes [18-21]. Although these prediction models show high confidence levels, they still have two limitations: first, the conserved orthologs between species only account for a small portion of the genome [22] and, second, the orthologs, especially in distantly related species, often show variations in gene regulations and functions [6,23], which lead to potential diversity in gene essentiality. To circumvent these limitations, feature-based models have been constructed to distinguish essential genes from non-essential ones based on common or similar features among essential genes [24-28].

In previous models, feature selection was often based on significant correlations between gene essentiality and gene features or the significant distribution difference between essential and non-essential genes [29-31]. A common disadvantage of such selection method, however, is that feature–feature interactions and strong correlations among features are ignored [32]. Moreover, because of evolutionary divergence among species, the linkages between features and gene essentiality might have changed. For example, arguments on whether or not younger genes are less likely to be essential than older genes [33,34] or whether or not duplicate genes are less likely to be essential than singletons [34-36], demonstrate that gene essentiality associations with origin time and number of duplications are diverse among different species.

Aside from feature selection, machine learning algorithms have also been introduced into feature-based classification models to identify essential genes in many studies, such as Naïve Bayes [25], decision tree [26], and support vector machine (SVM) [27].

In the present study, we first collected 16 features (see feature abbreviations in Table 1) that were widely used in previous models, and demonstrated that the predictions exhibit at least two problems: (1) strong correlations among gene features and (2) different and even contrasting associations of gene features with gene essentiality among different species. We then presented a novel approach, the feature-based weighted Naïve Bayes model (FWM), which can address multicollinearity impacts among gene features and feature divergence between species. In the proposed model, prior information was collected to determine the weight of each feature by logistic regression and genetic algorithm [37]. Afterward, essential genes in target organisms were predicted using a weighted Naïve Bayes (WNB) classifier [38]. We applied FWM to reciprocally predict essential genes between and within 21 species and compared its performance with those of other models including SVM, Naïve Bayes model (NBM), and logistic regression model (LRM). Results showed that FWM can significantly improve the accuracy and robustness of essential gene prediction. Finally, using stepwise discriminate analysis (SDA), we demonstrated why FWM outperforms these other classifiers.

**Table 1:** Abbreviations and descriptions of selected features

**Abbreviation**	**Description**
GO	Gene ontology
mE	mRNA expression level
mEF	mRNA express fluctuation
Age	Gene origin age
DoT	Gene domain type
DoC	Gene domain conservation
DC	Network topology feature, degree centrality
CCo	Network topology feature, clustering coefficient
CC	Network topology feature, closeness centrality
BC	Network topology feature, betweenness centrality
PL	Protein length
CAI	Codon adaptation index
NP	Number of paralogs for a target gene
NS	Number of species which have at least a homology for a target gene
NEH	Number of essential homolog genes in other species for a target gene
NNH	Number of non-essential homolog genes in other species for a target gene

## Results and discussion

### Relationship of gene features and gene essentiality

Selecting features associated with gene essentiality is fundamental to predict essential genes in feature-based models. However, because of the correlations between features, some features may actually share no or very few linkages with gene essentiality. Moreover, although feature linkages with gene essentiality exist, these linkages in different species are diverse or have contrasting effects.

To illustrate the possible consequences of different features in essential gene prediction, we investigate the linkages between gene essentiality and gene features in the *Saccharomyces cerevisiae* (*SCE*, Table 2A) and *Escherichia coli* (*ECO*, Table 2B) genomes, using the stepwise regression model (SRM) combined with forward selection [39-41]. At the beginning of the experiment, no features are considered in the model. A feature that mostly improves the model is added, and this process is repeated until all features are included in the model. The first column of Table 2 shows the results of the sequential addition of features into the SRM. Among the 12 features (Table 2A), the feature DC is the most important factor that explains the variation (6.5%) of gene essentiality. Some close neighboring features (e.g., NEH and NP) show statistical significance in terms of both correlations and true effects (i.e., standardized regression coefficients) with gene essentiality in the model. The last selected features (i.e., CAI, CC, and mE) also show statistical significance in linkage with gene essentiality; however, their true effects are detected without statistical significance (P-value > 0.05). This result may be explained by the fact that CC is highly correlated with DC (r = 0.765, P-value < 0.01), and DC has been selected as the first feature in the model that has diminished the effects of CC. One reason that may explain the lack of significant true effects exerted by CAI and mE is that both features have significant correlations with DC (r = 0.298, r = 0.393, and both P-values < 0.01) and CC (data not shown). Another explanation is that some essential genes show low expression levels. For example, the genes whose products are located in nuclear part (GO: 0044428) are overrepresented among the essential genes with lower expression levels. In addition, some transcription factors and centromere-associated proteins are only required in small amounts; however, these substances may be expressed constitutively and indispensably [42]. A similar pattern is observed during *ECO* analysis; however, compared with the *SCE* genome (Table 2A), the same features (e.g., NS) often show distinct effects on gene essentiality in the SRM (Table 2B). Most features in *SCE* contribute much less to gene essentiality than those in *ECO*. The genes in *SCE* are thus postulated to have more complicated and diverse functions than those in *ECO*, and the essentiality of these genes must be explained by a larger variety of features, which is expectedly in agreement with that eukaryotes are more complex than prokaryotes.

**Table 2:** **Linkages of features and gene essentiality in *SCE *** **(A) and *ECO *** **(B)**

**A**					
**Feature**	**Correlation**^**1**^	**P-value**^**2**^	**True effect**^**3**^	**P-value**^**4**^	**R Square**^**5**^
DC	0.256	3.7E-58	0.157	1.6E-09	0.065
NEH	0.188	5.5E-32	0.345	4.8E-61	0.087
NP	−0.054	4.5E-04	−0.186	2.5E-23	0.118
NS	0.001	0.47034	−0.225	8.0E-24	0.133
Age	−0.138	5.9E-18	−0.150	8.3E-15	0.150
CCo	0.166	4.0E-25	0.080	2.3E-06	0.155
DoC	0.132	1.9E-16	0.058	2.5E-04	0.157
mEF	−0.048	0.00166	−0.044	0.01224	0.159
CAI	0.043	0.00398	−0.033	0.07042	0.160
PL	0.020	0.10449	0.022	0.14817	0.161
CC	0.204	2.1E-37	0.021	0.40713	0.161
mE	0.086	5.4E-08	−0.009	0.65639	0.161
**B**					
**Feature**	**Correlation**	**P-value**	**True effects**	**P-value**	**R Square**
DC	0.486	6.E-228	0.368	1.2E-38	0.237
NEH	0.478	2.E-219	0.359	2.E-110	0.350
NNH	−0.148	1.7E-20	−0.327	3.4E-42	0.382
NS	0.283	6.7E-72	0.147	7.8E-11	0.390
CC	0.314	6.0E-89	−0.115	2.4E-06	0.396
NP	−0.151	2.9E-21	0.085	1.3E-05	0.399
mEF	0.147	2.7E-20	−0.058	6.9E-05	0.401
CAI	0.302	3.7E-82	0.024	0.11874	0.402
mE	0.266	1.4E-63	0.036	0.02500	0.402
PL	0.038	0.00940	0.024	0.07566	0.403
DoC	0.106	1.8E-11	0.013	0.32555	0.403
CCo	0.250	3.0E-57	0.004	0.79730	0.403

Note: The features from top to bottom in Column 1 are shown in accordance with the sequence in which they are added to the SRM. Data for gene essentiality in *SCE* and *ECO* originate from the relative growth rates of single-gene deletion yeast strains in nutrition-rich YPD medium [43] and *E. coli* chromosome (PEC) database profiling [44], respectively. Pearson correlations^1^ between gene essentiality and features are shown with a corresponding P-value^2^. True effect^3^ refers to the standardized regression coefficient in the model and it is presented with a related P-value^4^. R Square^5^ represents the ratio of the variance predicted by the SRM and the variance for gene essentiality.

If excessive features that contributed less to the model were selected, the process would inevitably lead to a complex and inefficient regression model. Besides, the same feature can result in different or opposite effects in different species, (e.g., NS has opposite effects in *ECO* and *SCE*). Therefore, selection of improper or excessive features may lead to redundancy and decrease the accuracy of the essential gene prediction model. These effects contradict the original goal of the essentiality analysis. In the current study, to overcome the deficiency in essential gene prediction, we developed a new method called FWM.

### FWM construction

**Figure 1:**
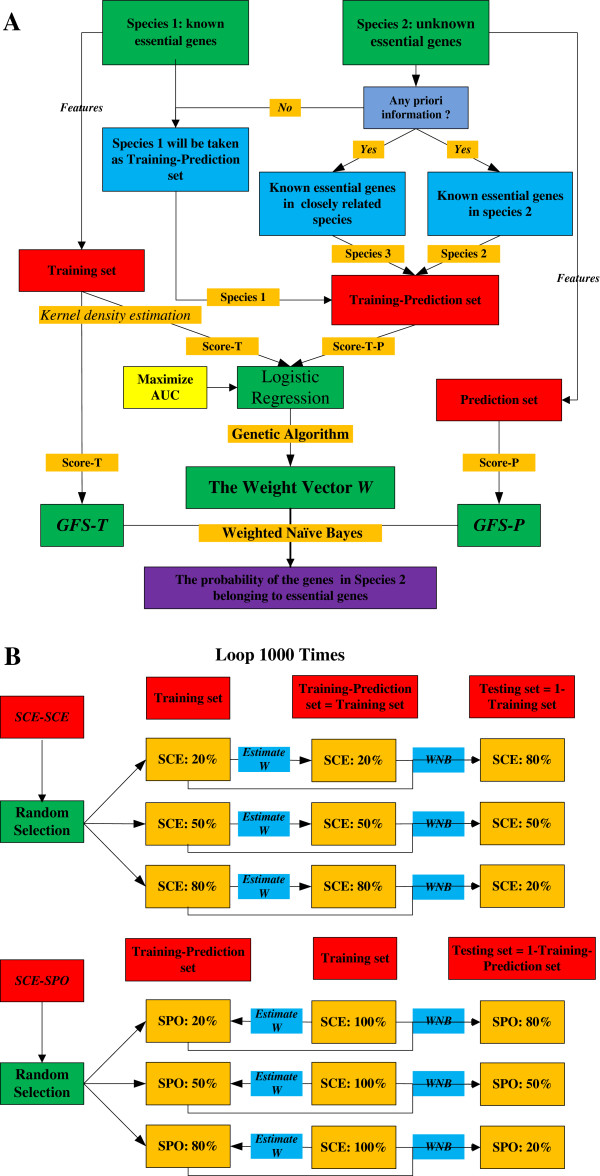
**Flow chart for constructing FWM and assessing its performance in predicting essential genes between and within species. (A)** FWM construction. During essential gene prediction from species 1 to species 2, the goal of FWM is to calculate the score vector *S*_*i*_ and the weighted coefficient vector *W*. To calculate *S*_*i*_, we mainly employ kernel density estimation (KDE) combined with Naïve Bayes estimation (see Methods). When calculating *W*, we first collect prior information (e.g., known essential genes in species 2 or from a closely related species); this information is used as training-prediction dataset to assess *W* in combination with the training set. Finally, we calculate the posterior probability of the genes in species 2 belonging to essential genes based on the weighted Naïve Bayes (WNB) method. **(B)** FWM performance for predicting essential genes between and within species. To assess the performance of FWM within species (e.g., *SCE*–*SCE* or *SPO*–*SPO*), 20%, 50%, and 80% of the whole genes were randomly selected as the training set, respectively, and the rest as testing set. We used the training set itself as a training-prediction set to calculate weights; the AUC score for the testing set was then calculated through the WNB method. Finally, the process was replicated 1,000 times to obtain the corresponding AUC distributions. To predict essential genes between species (e.g., *SCE*–*SPO* or *SPO*–*SCE*), all of the genes in *SCE* (or *SPO*) were selected as the training set, 20% (or 50%, 80%) of the *SPO* (or *SCE*) genes were randomly selected as the training-prediction set, and the rest of the genes were designated as the testing set. Similar to the comparison within species, AUC distributions were obtained by replicating the process 1,000 times.

Among the various classification methods, the Naïve Bayes classifier [45] is a simple, fast, and efficient algorithm. Thus, Naïve Bayes classifiers are widely used in identifying essential genes, disease genes, and housekeeping genes [25,28,46-48]. In the current study, we developed another method called FWM (Figure 1A) that effectively addresses the effects of multicollinearity among gene features in NBM and overcomes the disadvantages of training and prediction sets with equal global feature score (*GFS*, see Appendix).

The basic FWM formula in the present study is described below (see inference in Appendix). For one gene *g*_*i*_(*g*_*i*_ ∈ *G*, *i* = 1, 2, ⋯, *m*) with a feature vector *X*_*i*_, the probability of the gene belonging to the class *E* (*E* = essential) is:

(1) $$P\left(g_{i}\in E|X_{i}\right)=\frac{1}{1+e^{-S_{i}\cdot W}}$$

where *W* is the weight vector indicating the extent of contribution of the features to gene essentiality and *S*_*i*_ is the feature score vector corresponding to logarithmic likelihood ratio: $S_{i}=\log\frac{P\left(X_{i}|g_{i}\in E\right)}{P\left(X_{i}|g_{i}\in \bar{E}\right)}$.

The key FWM procedure is the evaluation of *S*_*i*_ and *W*. For *S*_*i*_ calculation, our selected features are divided into continuous type (e.g., protein length) and non-continuous type (e.g., domain type); we then employ kernel density estimation [49] and Bayes estimation to calculate the *S*_*i*_ of these two types of features (see Methods).

To evaluate *W*, we need a *W* that can reflect the true contribution of the features in the target species. Therefore, we first determine some prior information based on a known essential gene set, which is preferably from the target species or a species that is closely related to the target species. Note that we define the known essential gene set as the training-prediction set used as a dependent variable to help evaluate *W*. If we cannot obtain any prior information, the training set is also used as an alternative training-prediction set to calculate *W*. According to formula (1), we obtain $S_{i}\times W=\log\frac{P\left(g_{i}\in E|X_{i}\right)}{1-P\left(g_{i}\in E|X_{i}\right)}$. We then imitate the estimation of logistic regression coefficients to calculate *W*. To obtain high essential gene prediction accuracy, we estimate the parameter *W = argmax*{*AUC*[*PP*(*W*)*, GE*]} using genetic algorithm. Here, *PP*(*W*) represents the posterior probability vector calculated by formula (1), *GE* represents the true gene essentiality determined from the training-prediction set, and the AUC (area under curve) score is calculated from *PP*(*W*) and *GE*. Finally, we calculate the posterior probability of the genes in Species 2 (the target species whose essential genes need to be predicted) based on the WNB method again using formula (1).

### FWM accuracy, stability, and adaptability

Because FWM is developed from NBM, we first compared the predictive performances of FWM and NBM within and between species. Two eukaryotic species (*SCE* and *Schizosaccharomyces pombe* (*SPO*)) that have well-characterized essential genes were used as either training sets or testing sets (Figure 1B).

**Figure 2:**
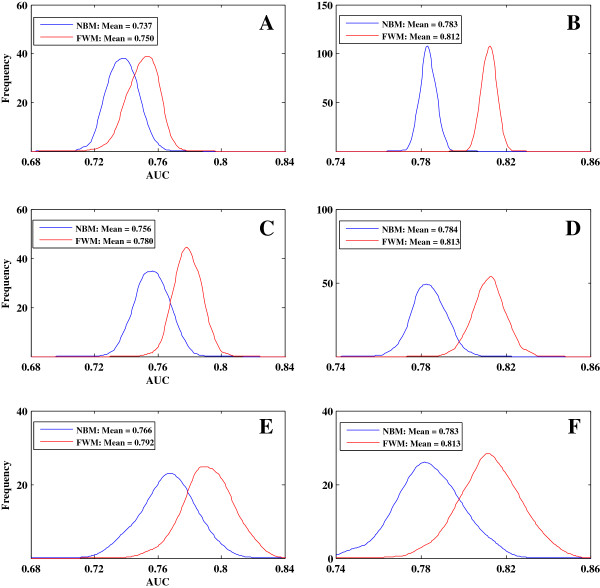
**Essential gene prediction within and between species by NBM and FWM. A**, **C**, and **E** show the AUC distributions within species (*SCE*–*SCE*), which are generated by randomly selecting 20% **(A)**, 50% **(C)**, and 80% **(E)** of the *SCE* genes as training data. **B**, **D**, and **F** show the AUC distributions between species (*SCE*–*SPO*), which are generated by randomly selecting 20% **(B)**, 50% **(D)**, and 80% **(F)** of the *SPO* genes as a training-prediction set to estimate the weight vector *W*. Blue and red lines represent the distributions obtained by NBM and FWM, respectively.

To investigate the accuracy and stability of FWM, 20% of the *SCE* genes were randomly selected as both the training set and the training-prediction set to help calculate *W*; the rest of the genes were used as the testing set. FWM and NBM were then used to predict and calculate AUC scores, respectively. The simulation was replicated 1,000 times (randomly selected training set and testing set), and two corresponding AUC distributions were obtained (Figure 2A). Similarly, 50% and 80% of the genes in *SCE* were respectively randomly selected and simulated with 1,000 replications to obtain the AUC distributions (Figures 2C and E). By comparing the AUC distributions obtained by FWM and NBM, we found that the mean values from FWM were significantly higher than from NBM (*T*-test and P-value < 1e-100; Figure 2A, C and E). This finding demonstrates that the results within species predicted by FWM are more accurate than those predicted by NBM. Similar results were obtained in *SPO* (Additional file 1: Figure S1-A, S1-C and S1-E).

To assess the adaptability of FWM between different species, we adopted the strategy to predict essential genes in *SPO* based on training dataset from *SCE*. First, 20%, 50%, and 80% of the *SPO* genes were randomly selected as training-prediction sets (the rest of the genes were used as the testing set), and the corresponding weight vector *W* was obtained. We then predicted the remaining set of *SPO* genes using *W* and the training set from *SCE*. Finally, we obtained the AUC distributions with 1,000 replicated simulations. The results were shown in Figures 2B (20%), 2D (50%), and 2F (80%). A similar analysis was performed in the prediction from *SPO* to *SCE* (Additional file 1: Figures S1-B, S1-D, and S1-F). Consistent with the results of prediction within species, FWM showed better performance than NBM for predictions between species. In addition, while obtaining an accurate vector *W*, FWM easily reaches a saturation point when some prior information is supplied.

### Comparison of FWM with LRM, NBM, and SVM

We applied FWM to 21 species (including 19 bacteria and two fungi; listed in Additional file 2: Table S1) to illustrate: (1) the validity of FWM predictions of essential genes in diverse species and (2) the advantages of FWM over other methods. The genes in the 21 species were taken in turns to use as training and testing sets. The process yielded a 21 × 21 AUC matrix represented as *M* = (*m*_*ij*_), *i,j* = 1, 2,…, 21, where *m*_*ij*_ indicates the AUC score obtained with *i*th species as training set and *j*th species as testing set.

**Figure 3:**
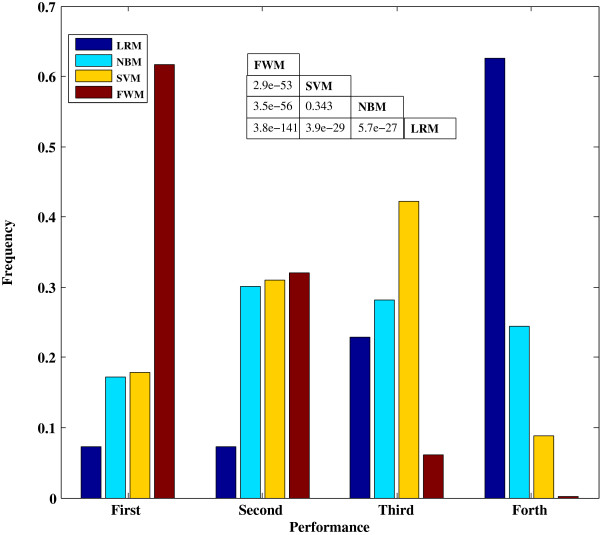
**Comparison of FWM with LRM, NBM, and SVM.** Four AUC matrices among the 21 species are produced using the four methods. AUC scores (*m*_*ij*_) in the same position of the four matrices are then sorted and replaced with markers (first with the maximum AUC score, followed by the second and third, and, finally, fourth with the minimum score) for the four methods. By calculating the frequency of the ranking list (i.e., first, second, third, and forth) in the four matrices, performance distributions for the four methods were generated. The AUC score with ranking the first and second can be classified as high-quality performance, while third and fourth can be classified as low-quality performance. Significant differences are tested by Fisher’s exact test, and the results are shown in the lower triangular table.

The accuracy of FWM prediction was compared with three other classifiers: LRM, NBM, and SVM. Each of these classifiers yielded a 21 × 21 matrix with a total of 441 AUC scores (Additional file 2: Table S2) independently. Afterward, we sorted the AUC scores of variable *m*_*ij*_ produced by the four approaches (Figure 3, see details in Additional file 2: Table S3). 61.7% (272/441) AUC scores produced by FWM were ranked in first tier (which represented the AUC score is the maximum among the quadruple AUC scores generated by the four methods) and only one was located in the fourth tier (the AUC score is the minimum among the quadruple AUC scores). Evidently, FWM significantly outperformed the other three methods (P-value < 1e-53). By the way, the performance of SVM was slightly but not significantly better than that of NBM (P-value = 0.343). Although the performance of LRM was the worst among the four approaches studied, this model showed strong overfitting, which can lead to better performance during cross-validation within species than NBM and SVM. Anyhow, our method is substantially superior to LRM, NBM, and SVM for predicting essential genes.

### Why does our approach perform better prediction effects?

**Figure 4:**
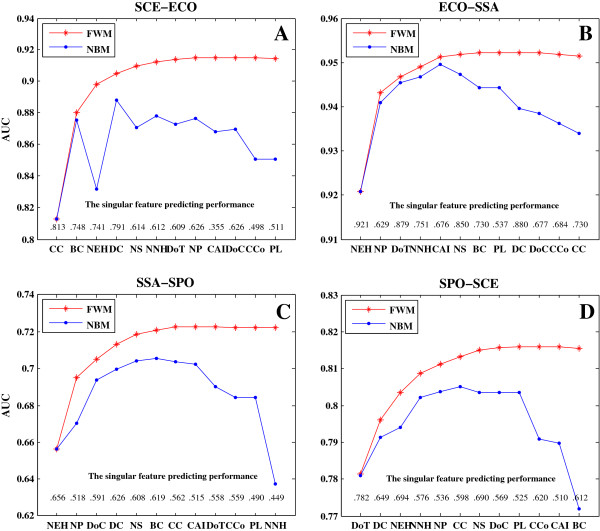
**Comparison of FWM and NBM in stepwise discriminant.** Examples of *SCE–ECO***(A)**, *ECO–SSA***(B)**, *SSA–SPO***(C)**, and *SPO–SCE***(D)** are plotted. The labels on the X-axis from the left to right indicate the order of the features selected into the model according to their prediction effects. The values above the X-axis represent the singular prediction effect of the corresponding feature. FWM indicates feature-based weighted Naïve Bayes model and NBM indicates Naïve Bayes model.

To explain why FWM outperforms the other classifiers, we employed SDA combined with forward selection [50] to investigate AUC variations; FWM and NBM were used as discriminate models. During the repeating selection process, the features that could improve AUC score the most in the classifiers were selected one by one, until all of the features were included in the model. Four microbes with well-characterized essential genes were used in our analysis (Figure 4), including two bacteria (i.e., *ECO* and *Streptococcus sanguinis* (*SSA*)) and two fungi (i.e., *SCE* and *SPO*). The labels on the X-axis from left to right in Figure 4 indicated the order of the features selected into the model one by one. During analysis, the following features showed the best performance: CC in *SCE–ECO*, NEH in *ECO–SSA* and *SSA*–*SPO*, and DoT in *SPO–SCE*. The best performance of CC demonstrated that essential genes tend to play topologically more important roles in protein interaction networks than non-essential genes. *SCE* and *ECO* had more complete and available interaction data than the other organisms; thus, CC neither performs the best among the three other predictions. The best performance of NEH showed that orthologous gene essentiality is conserved across organisms. DoT had the best performance in *SPO–SCE* prediction because, relative to that in bacteria, gene essentiality is more conserved through the function of protein domains or domain combinations rather than through the conservation of the entire genes in fungi [28].

Although the feature GO has a better effect than other features in the prediction (see Additional file 2: Table S4), most genes with known GO annotations have only been recorded in well-studied model species but have not been investigated in the non–model organisms. Thus, selecting GO as the feature to predict essential genes in a non–model or a new sequenced organism is inappropriate. Values close to the X-axis (Figure 4) indicate the singular prediction effect of the corresponding feature. Except for the first feature, the selection order of other features into the prediction model was based on the diminished effect from the previous selected features. For example, in the prediction from *SPO* to *SCE*, the AUC score generated by a single NS feature is 0.69, which ranks third among all of the features; but this feature was selected as the seventh feature in SDA because of the partial replacement of NS effects by previously selected features.

FWM performed better than NBM in all cases. The prediction accuracy reached a saturation point when some key features were selected into the model. The NBM classifier substantially reduced the prediction accuracy at the end of prediction, whereas FWM provided redundant features with small weights to avoid such a problem (see Additional file 2: Table S5) and showed slow changes in prediction accuracy with addition of features.

**Figure 5:**
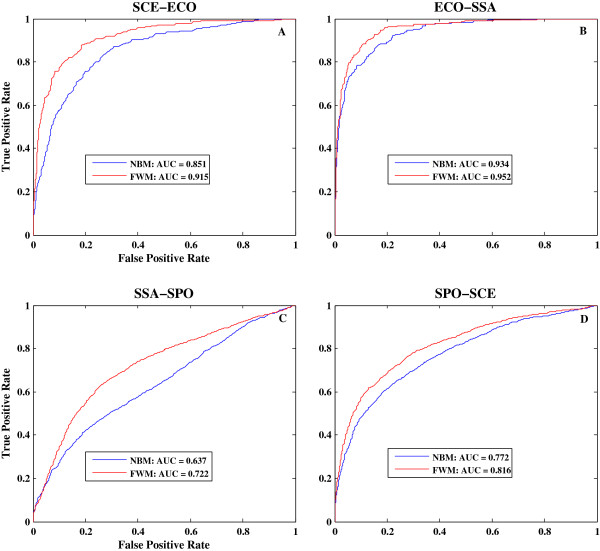
**Comparison of ROC curves between FWM and NBM.** Examples of *SCE–ECO***(A)**, *ECO–SSA***(B)**, *SSA–SPO***(C)**, and *SPO–SCE***(D)** are plotted. TPR (sensitivity) is plotted on the Y-axis and FPR (1-Specificity) is plotted on the X-axis with threshold values from 0 to 1. Blue lines represent ROC curves generated by NBM and red lines represent ROC curves generated by FWM. FWM indicates feature-based weighted Naïve Bayes model, NBM indicates Naïve Bayes model, and AUC indicates area under curve.

In Figure 5, we compared receiver operating characteristic (ROC) curves generated by FWM and NBM in four microbes. FWM consistently showed a significant higher true positive rate (TPR) and a significantly lower false positive rate than NBM in all four predictions, except for the location of ROC curves at approximately 0 or 1 in the X-axis. The increases in AUC based on FWM relative to NBM in *SCE–ECO*, *ECO–SSA*, *SSA–SPO*, and *SPO–SCE* are 0.064, 0.018, 0.085, and 0.044, respectively. AUC score also indicated the average TPR in all threshold values [51]; thus, our FWM could improve prediction accuracy at least from 2% to 9%. In general, FWM provides a more effective way of integrating features associated with gene essentiality, and overcomes the impact of multicollinearity among features. Therefore, FWM presents the advantages of increased adaptability and reliability for essential gene prediction.

## Conclusions

Selecting features associated with gene essentiality is necessary to identify essential genes through machine learning approaches. However, current studies often neglect the phenomenon of multicollinearity among these features, and the same feature may result in different and even contrasting effects among species. Selecting such features makes the prediction model cumbersome, does not improve prediction precision, but contrarily, may decrease the accuracy of essential gene prediction. In other words, selecting more features does not mean better prediction results.

To address these problems, we built FWM by improving the Naïve Bayes classifier. This new model assigns a corresponding weight for each feature based on its real contribution to the model, and importantly, this weight can be changed depending on the specific target organisms. FWM is able to effectively alleviate the effects of both multicollinearity among features and the complex relationship between features and gene essentiality in different organisms. In summary, FWM is able to improve predictive accuracy compared with other methods (e.g., NBM, LRM, and SVM).

Xu et al. [52] revealed that essential genes are associated with only three basic categories of essential functions or processes in organisms: cell envelope maintenance, energy production, and genetic information processing. Nevertheless, the genes engaged in essential functions may yield a conditional essential gene during evolution [3,7]. Others may be compensated by a duplicate or buffered by some new metabolic network flux reorganization that results in the transformation of essentiality in the original reaction or path. Besides, because of changes in the external environment or evolution from lower to higher organisms, many new essential functions and metabolic processes can emerge. Thus, gene essentiality constantly changes over time and more efforts are needed to completely understand the minimal requirements for cellular life. In the current study, we presented a theoretical frame and a practical strategy to predict mass genome-wide essential genes. Our method reduced the burden of systematic understanding of the minimal requirements for cellular life and can help identify potential drug targets in novel pathogens.

## Methods

### Essential gene and gene sequences

The essential genes of 21 species (see the species in Additional file 2: Table S1 and the collected essential gene in Additional file 3) were obtained from relevant studies, as well as the Online Gene Essentiality Database (OGEE) [53] and Database of Essential Genes (DEG) [54]. The cDNA and protein sequences of the 21 species were downloaded from the NCBI server (ftp://ftp.ncbi.nih.gov/genomes/). The homologous map and proteome sequences of 417 core species were downloaded from eggNOG 3.0 [55].

### Features collection

We collected 16 features (Table 1), 12 of which were widely used in previous models and 4 of which were used for the first time in our model (see details for the 16 features in Additional file 4: Table S6). The distribution difference of each feature between essential and non-essential genes is shown in Additional file 4: Figures S2-S13.

(i) Domain and GO annotations. Essential genes are associated with basic categories of biological functions or processes [52]. Therefore, essential genes may enrich some domains or GO annotations. To collect the domain of each gene in 21 species, the hidden Markov models (Pfam-A.hmm) of the protein domains were downloaded from the Pfam database [56], and Hammer [57] was used to identify the protein domain for each gene. The corresponding domain type for each gene (see details of identifed domains for the 21 species in Additional file 5: Table S7) was defined as the feature DoT. The amino acid sites within protein domains are often more important and conserved than other fractions. Therefore, we assumed that the protein domain conservation is a reflection of gene essentiality, and the DoC of each gene was calculated from the ratio of the conserved domain score and the domain length. GO annotations were downloaded from the Gene Ontology Database [58]. GO enrichment analysis of *SCE* and *SPO* is shown in Additional file 5: Table S8.

(ii) Protein–protein interaction (PPI) network. Network topology features have been widely used in previous papers (Additional file 4: Table S6). In our study, PPI data for the genes in 21 species were downloaded from the STRING Database [59]. Afterward, we used the NetworkX software package [60] to compute the four network topology features of DC, CCo, CC, and BC.

(iii) Genomic sequence properties. Although protein length (PL) tends to become longer through evolution [61], different natural constraints might exist on the PL between essential genes and nonessential genes. The codon usage of essential genes suffers from more evolutionary constraints than non-essential genes. We used the CodonW [62] software package to calculate the codon usage, i.e., CAI.

(iv) Homology properties. Duplicated genes are believed to often overlap in function and expression [63], and duplicates are always less likely to be essential than singletons [34,64,65]. An all-against-all BLAST search was conducted for the whole set of proteins in each of 21 species to identify the paralogs with an E-value threshold of 10^-20^, and the number of paralogs for a target gene within species was used as the feature NP. Four-hundred seventeen core organisms in the eggNOG database included all of 21 species in our study; thus, we counted the number of species among the 417 core species that had at least one homologous gene for each target gene in 21 species (feature NS). The orthologous gene of an essential gene is highly likely to be essential as well [18]. Therefore, we calculated the numbers of essential and non-essential homologous genes, including those that are found in other species, for each target gene (NEH and NNH).

(v) Phyletic gene age. Chen [34] showed that older genes (i.e., genes with earlier phyletic origin) are more likely to be essential than young ones. Age was calculated according to previously described methods [34,66]; the target genomes of *SCE* and *SPO* were divided into five taxonomic groups, i.e., species typical, Ascomycota, Opisthokonta, Eukaryota, and cellular organisms.

(vi) Gene expression. mRNA expression data were obtained from Series GSE15352 [67] and GSE30025 [68] of the Gene Expression Omnibus (GEO) Database. The expression levels of essential genes are often generally higher and more stable than those of non-essential genes [69]. The average and variable coefficients of mRNA expression levels in all conditions were collected as predictors (i.e., mE and mEF).

### Calculation of the feature score vector S_ij_

Features can be classified into two types: continuous and non-continuous. For continuous features, the kernel density estimation [KDE; the estimate is implemented by MATLAB’s “ksdensity” function, using a normal kernel function and a window parameter (bandwidth) that is a function of the number of points in the sample] [49] is employed to acquire the empirical probability density function *f (x|E)* for essential genes and $f\left(x|\bar{E}\right)$ for non-essential genes [49]. The feature score vector *S*_*ij*_ can be calculated as $S_{\mathrm{ij}}=\log\frac{f\left(x|E\right)}{f\left(x|\bar{E}\right)}$. For non-continuous features, the analysis is much more complicated (see Additional file 6). In the current study, we only displayed the inferred result: $S_{\mathrm{ij}}=\log\frac{\left(n_{\mathrm{jE}}+1\right)/\left(N_{E}+2\right)}{\left(n_{j\bar{E}}+1\right)/\left(N_{\bar{E}}+2\right)}$, where *n*_*jE*_ and $n_{j\bar{E}}$ indicate the number of essential and non-essential genes, respectively, sharing the same value for a given feature, and *N*_*E*_ and $N_{\bar{E}}$ indicate the total number of essential and non-essential genes, respectively.

### Other classifiers

We compared three classifiers with FWM: (1) NBM, (2) LRM, and (3) SVM. Each classifier scheme independently generates a separate probability score of gene essentiality. All classifiers were implemented using the Weka software package [70]. The outline of Weka procedures with JAVA codes is shown in below.

**Input:** Attribute relation function format (ARFF) files of feature data of 21 organisms.

Parameters:

1. Sequential minimal optimization (SMO) (weka.classifiers.functions. SMO -C 1.0 -L 0.001 -P 1.0E-12 -N 0 -M -V −1 -W 1 -K “weka.classifiers.functions.supportVector.RBFKernel -C 250007 -G 0.01”)

2. NaïveBayes (default settings)

3. Logistic (weka.classifiers.functions. Logistic -R 1.0E-8 -M −1)

**Output:** 21 × 21 AUC matrices of SVM, NBM, and LRM

1. Read the training and testing sets

2. For each species as training set

2.1. Build classifiers NaiveBayes(), Logistic() and SMO()

2.1. For each species as testing set

2.2. Evaluate each classifier

2.2. Write evaluation.toSummaryString(), evaluation. toClassDetailsString(), evaluation.toMatrixString()2.2.3 Extract ROC area score

3. For each classifier

3.1. Arrange ROC area scores to the matrix

### Appendix: The derivation of formula (1) for FWM construction

In Naïve Bayes algorithm, the probability that gene *g*_*i*_(*i* = 1, 2, ⋯, *m*) belongs to class *E* (*E* = essential and $\bar{E}=\mathrm{non}-\mathrm{essential}$) given the feature vector *X*_*i*_ = [*x*_*i*1_, *x*_*i*2_, ⋯, *x*_*in*_] is:

(2) $$P\left(g_{i}\in E|X_{i}\right)=\frac{P\left(E\right)\prod_{j=1}^{n}P\left(x_{\mathrm{ij}}|g_{i}\in E\right)}{P\left(X_{i}\right)}$$

where *j* indicates the *j*th feature in all *n* features for gene (*g*_*i*_); *P*(*E*) indicates the prior probability of a gene belonging to an essential gene (in general, *P*(*E*) is represented by the proportion of essential genes in all genes); *P*(*x*_*ij*_|*g*_*i*_ ∈ *E*) denotes the conditional probability when we observe that the *j*th feature value is *x*_*ij*_ under the condition that the *i*th gene (*g*_*i*_) is an essential gene; and *P*(*X*_*i*_) is from the complete probability formula:

$$P\left(X_{i}\right)=P\left(E\right)\prod_{j=1}^{n}P\left(x_{\mathrm{ij}}|g_{i}\in E\right)+P\left(\bar{E}\right)\prod_{j=1}^{n}P\left(x_{\mathrm{ij}}|g_{i}\in \bar{E}\right)$$

We obtain:

(3) $$P\left(g_{i}\in \bar{E}|X_{i}\right)=\frac{P\left(\bar{E}\right)\prod_{j=1}^{n}P\left(x_{\mathrm{ij}}|g_{i}\in \bar{E}\right)}{P\left(X_{i}\right)}$$

We use the ratio of (2) and (3) and set:

$$P\left(g_{i}\in \bar{E}|X_{i}\right)=1-P\left(g_{i}\in E|X_{i}\right),P\left(\bar{E}\right)=1-P\left(E\right)$$

Finally, we obtain:

(4) $$P\left(g_{i}\in E|X_{i}\right)={\left[1+e^{-\log\frac{P\left(E\right)}{1-P\left(E\right)}-\sum_{j=1}^{n}S_{\mathrm{ij}}}\right]}^{-1},$$

where $S_{\mathrm{ij}}=\log\frac{P\left(x_{\mathrm{ij}}|g_{i}\in E\right)}{P\left(x_{\mathrm{ij}}|g_{i}\in \bar{E}\right)}$ indicates the logarithmic likelihood ratio, which we refer to as the feature score. We define the $\mathrm{GFS}=\sum_{j=1}^{n}S_{\mathrm{ij}}$ as the global feature score. If we suppose that *GFS* is a function of the feature vector *X*_*i*_, the Naïve Bayes classifier comes into existence based on the fundamental conditions that the features must be mutually independent and that the training and prediction sets must have the same *GFS* function.

Unfortunately, the assumption of mutual independence for NBM does not always hold true, and training and prediction sets will not always have the same *GFS* function. To solve the problems, we add a weighted coefficient *w*_*j*_ prior to *S*_*ij*_. The global feature score is redefined as $\mathrm{GFS}=\sum_{j=1}^{n}w_{j}S_{\mathrm{ij}}$, and

(5) $$P\left(g_{i}\in E|X_{i}\right)={\left[1+e^{-\log\frac{P\left(E\right)}{1-P\left(E\right)}-\sum_{j=1}^{n}\left(w_{j}S_{\mathrm{ij}}\right)}\right]}^{-1}$$

where *w*_*j*_ indicates the extent of the contribution of the *j*th feature to a gene classified as an essential gene. To simplify the model, we set $w_{0}=\log\frac{P\left(E\right)}{P\left(\bar{E}\right)}$, *S*_*i0*_ = 1, *S*_*i*_ = [*S*_*i*0_, *S*_*i*1_, *S*_*i*2_, ⋯, *S*_*in*_] and *W* = [*w*_0_, *w*_1_, *w*_2_, ⋯, *w*_*n*_]. The probability of a gene *g*_*i*_ belonging to essential gene is given by:

$$P\left(g_{i}\in E|X_{i}\right)=\frac{1}{1+e^{-S_{i}\cdot W}}.$$

We put the scripts for FWM construction and usage in Additional file 7.

## Competing interests

The authors declare that they have no competing interests.

## Authors’ contributions

JC conceived and designed the method. JC and WW performed the analyses. WW wrote the manuscript. All authors have read and approve of the final manuscript.

## Supplementary Material

Additional file 1: Figure S1 Essential gene prediction within and between species by NBM and FWM. AUC distributions within species (*SPO* - *SPO*) were generated by randomly selecting 20% (A), 50% (C) and 80% (E) of *SPO* genes as training data and training-prediction set, respectively. Whereas AUC distributions between species (*SPO*- *SCE*) were generated by randomly selecting 20% (B), 50% (D), and 80% (F) of *SCE* genes as training-prediction set to estimate weight vector *W*, respectively. The blue and red lines represent the distribution obtained by NBM and FWM, respectively. Click here for file

Additional file 2: Table S1 The fundamental information of 21 species. This table displayed the evolutionary relationships of 21 species, the experimental methods for identification of essential genes, and the growth conditions. **Table S2.** The AUC matrices of four methods. Each matrix was obtained with the corresponding method. The AUC score in the same position of matrices had the same training set and prediction set. **Table S3.** The AUC order matrices of four methods. The AUC scores (*m*_*ij*_) in the same position of the four matrices were sorted and replaced with numbers (1 was equivalent to the maximum AUC score, followed by 2 and 3, and 4 corresponded to the minimum score). We then calculated the frequency of the ranking list (i.e., 1, 2, 3, and 4) in the four matrices. The method which the corresponding matrix had more 1 or 2 was considered better method. **Table S4.** The AUC scores with singular feature in the predictions. **Table S5.** Comparing FWM and NBM in stepwise discriminant analysis. We employed SDA to investigate AUC variations by adding features one by one. The ‘Features’ column indicates the sequence into the model. The ‘Singular AUC Score’ column indicates the AUC score generated with only a feature in the model. The ‘Ranking’ column indicates the sorting of singular AUC score. The ‘*W*’ column indicates the weights of the corresponding feature in FWM. The last two columns indicate AUC variations with the additions of features). Click here for file

Additional file 3 **Essential genes dataset.** We collected currently available essential genes among a wide range of organisms, including 11 eukaryotes and 21 prokaryotes. Click here for file

Additional file 4: Table S6 Introduction and reference about our selected features. **Figure S2.** The distribution difference of DoC. **Figure S3.** The distribution difference of DC. **Figure S4.** The distribution difference of CCo. **Figure S5.** The distribution difference of CC. **Figure S6.** The distribution difference of BC. **Figure S7.** The distribution difference of PL. **Figure S8.** The distribution difference of CAI. **Figure S9.** The distribution difference of NP. **Figure S10.** The distribution difference of NS. **Figure S11.** The distribution difference of NEH. **Figure S12.** The distribution difference of NNH. **Figure S13.** The distribution difference of mE, mEF, and Age in *SCE* and *SPO*. Click here for file

Additional file 5: Table S7 Comparison of domain enrichment between the 19 bacteria and 2 fungi. We calculate the P-value using the hypergeometric distribution, and Bonferroni method is used for multiple hypothesis correction. **Table S8.** Comparison of Gene Ontology term enrichment between *S. cerevisiae* and *S. pombe*. We calculate the P-value using the hypergeometric distribution, and Bonferroni method is used for multiple hypothesis correction. Click here for file

Additional file 6 **Calculation of the score vector *S***_***i***_**for non-continuous features.** Click here for file

Additional file 7 FWM script. Click here for file
