# Supplementary figures and images for: A new computational strategy for predicting essential genes
Source: BMC Genomics. 2013 Dec 21;14:910. doi: 10.1186/1471-2164-14-910 (PMC3880044; doi:10.1186/1471-2164-14-910)

Figure S1

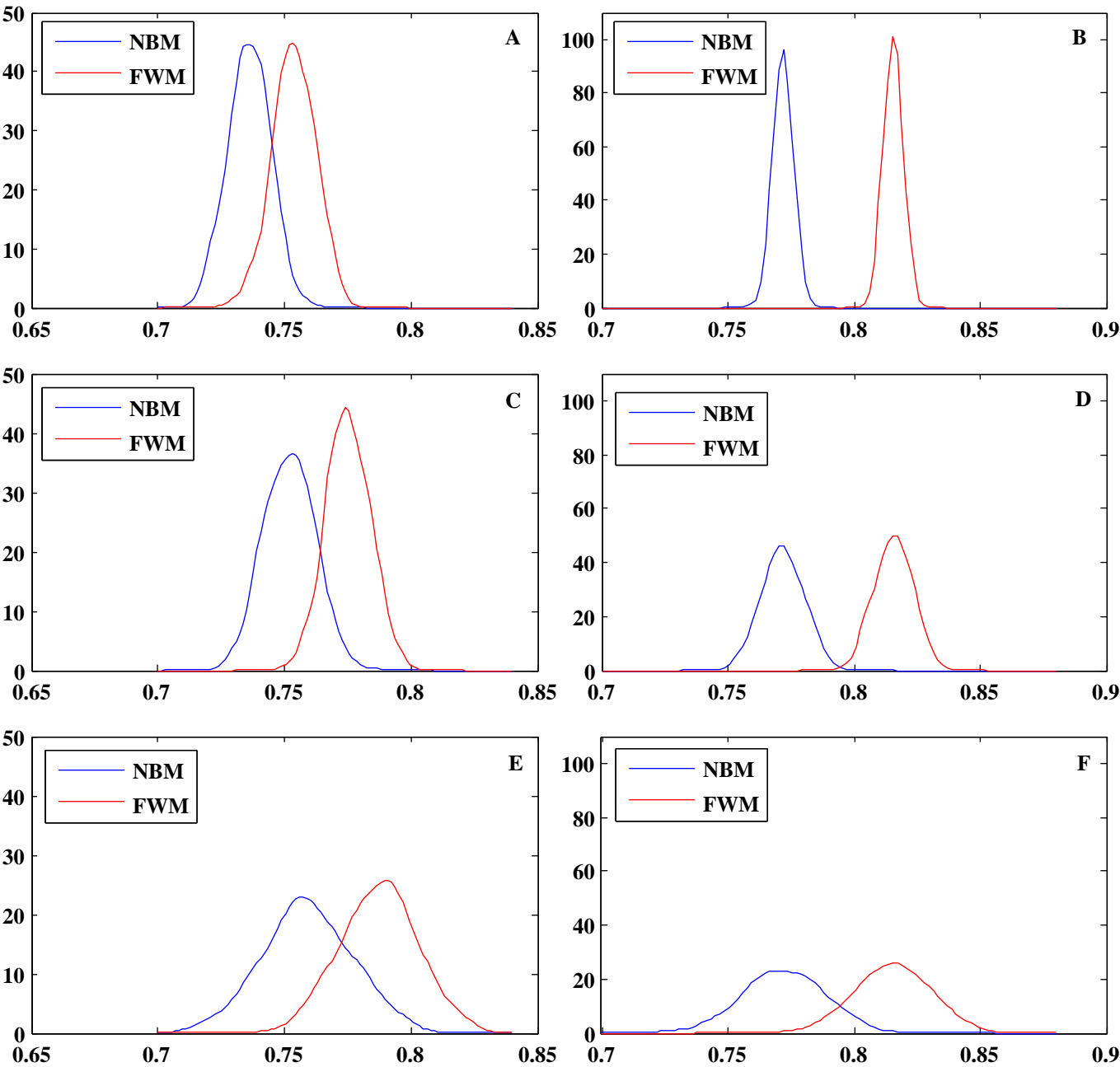

Supplement: Additional file 1: Figure S1 — Essential gene prediction within and between species by NBM and FWM. AUC distributions within species (SPO - SPO) were generated by randomly selecting 20% (A), 50% (C) and 80% (E) of SPO genes as training data and training-prediction set, respectively. Whereas AUC distributions between species (SPO- SCE) were generated by randomly selecting 20% (B), 50% (D), and 80% (F) of SCE genes as training-prediction set to estimate weight vector W, respectively. The blue and red lines represent the distribution obtained by NBM and FWM, respectively. [file 1471-2164-14-910-S1.pdf]
