# Supplementary material for: A new computational strategy for predicting essential genes
Source: BMC Genomics. 2013 Dec 21;14:910. doi: 10.1186/1471-2164-14-910 (PMC3880044; doi:10.1186/1471-2164-14-910)
Supplement: Additional file 4: Table S6 — Introduction and reference about our selected features. Figure S2. The distribution difference of DoC. Figure S3. The distribution difference of DC. Figure S4. The distribution difference of CCo. Figure S5. The distribution difference of CC. Figure S6. The distribution difference of BC. Figure S7. The distribution difference of PL. Figure S8. The distribution difference of CAI. Figure S9. The distribution difference of NP. Figure S10. The distribution difference of NS. Figure S11. The distribution difference of NEH. Figure S12. The distribution difference of NNH. Figure S13. The distribution difference of mE, mEF, and Age in SCE and SPO. [file 1471-2164-14-910-S4.doc]

**Table S6. Introduction and reference about our selected features.** We collected 16 features and 12 of them are widely used by previous papers. The DoC, NEH, NNH and Age are first used to predict essential genes in this study. The former 14 features were collected for all 21 species. The GO, mE, mEF, and Age were collected only for *S. cerevisiae* and *S. pombe*.

| **Abbreviation** | **Description** | **Reference** |
| --- | --- | --- |
| DoT | gene Domain Type |  |
| DoC | gene Domain Conservation | First use |
| DC | network topology feature, Degree Centrality |  |
| CCo | network topology feature, Clustering Coefficient |  |
| CC | network topology feature, Closeness Centrality |  |
| BC | network topology feature, Betweenness Centrality |  |
| PL | Protein Length |  |
| CAI | Codon Adaptation Index |  |
| NP | The Number of Paralogs for a target gene |  |
| NS | The Number of Species which have at least a homologous genes for a target gene |  |
| NEH | The Number of Essential Homologous genes in other species for a target gene | First use |
| NNH | The Number of Non-essential Homologous genes in other species for a target gene | First use |
| GO | Gene Ontology |  |
| mE | mRNA Expression level |  |
| mEF | mRNA Express Fluctuation |  |
| Age | gene origin Age | First use |

**Reference**

1. Deng J, Deng L, Su S, Zhang M, Lin X, Wei L, Minai AA, Hassett DJ, Lu LJ: **Investigating the predictability of essential genes across distantly related organisms using an integrative approach**. *Nucleic acids research* 2011, **39**(3):795.

2. Deng J, Tan L, Lin X, Lu Y, Lu LJ: **Exploring the optimal strategy to predict essential genes in microbes**. *Biomolecules* 2011, **2**(1):1-22.

3. Gustafson AM, Snitkin ES, Parker SCJ, DeLisi C, Kasif S: **Towards the identification of essential genes using targeted genome sequencing and comparative analysis**. *Bmc Genomics* 2006, **7**(1):265.

4. Estrada E: **Virtual identification of essential proteins within the protein interaction network of yeast**. *Proteomics* 2006, **6**(1):35-40.

5. da Silva JPM, Acencio ML, Mombach JCM, Vieira R, da Silva JC, Lemke N, Sinigaglia M: ***In silico* network topology-based prediction of gene essentiality**. *Physica A: Statistical Mechanics and its Applications* 2008, **387**(4):1049-1055.

6. Hwang Y-C, Lin C-C, Chang J-Y, Mori H, Juan H-F, Huang H-C: **Predicting essential genes based on network and sequence analysis**. *Molecular BioSystems* 2009, **5**(12):1672-1678.

7. Plaimas K, Eils R, König R: **Identifying essential genes in bacterial metabolic networks with machine learning methods**. *BMC systems biology* 2010, **4**(1):56.

8. Seringhaus M, Paccanaro A, Borneman A, Snyder M, Gerstein M: **Predicting essential genes in fungal genomes**. *Genome research* 2006, **16**(9):1126.

9. Acencio ML, Lemke N: **Towards the prediction of essential genes by integration of network topology, cellular localization and biological process information**. *BMC Bioinformatics* 2009, **10**(1):290.

10. Liu Y-C, Chiu P-I, Huang H-C, Tseng VS: **Prediction of essential genes by mining gene ontology semantics**. In: *Bioinformatics Research and Applications.* Springer; 2011: 49-60.

11. Jeong H, Oltvai ZN, Barabási A-L: **Prediction of protein essentiality based on genomic data**. *ComPlexUs* 2002, **1**(1):19-28.

**Figure S2. The distribution difference of DoC.** Differences of average DoC between essential and nonessential genes in the studied 21 genomes. Blue and red bars indicate essential and nonessential genes respectively.

**Figure S3. The distribution difference of DC.** Differences of average DC between essential and nonessential genes in the studied 21 genomes. Blue and red bars indicate essential and nonessential genes respectively.

**Figure S4. The distribution difference of CCo.** Differences of average CCo between essential and nonessential genes in the studied 21 genomes. Blue and red bars indicate essential and nonessential genes respectively.

**Figure S5. The distribution difference of CC.** Differences of average CC between essential and nonessential genes in the studied 21 genomes. Blue and red bars indicate essential and nonessential genes respectively.

**Figure S6. The distribution difference of BC.** Differences of average BC between essential and nonessential genes in the studied 21 genomes. Blue and red bars indicate essential and nonessential genes respectively.

**Figure S7. The distribution difference of PL.** Differences of average PL between essential and nonessential genes in the studied 21 genomes. Blue and red bars indicate essential and nonessential genes respectively.

**Figure S8. The distribution difference of CAI.** Differences of average CAI between essential and nonessential genes in the studied 21 genomes. Blue and red bars indicate essential and nonessential genes respectively.

**Figure S9. The distribution difference of NP.** For each species, the left and right columns indicate nonessential and essential genes respectively. The blue bar indicates the frequency of the genes without paralogs in constrast to the genes with at least one paralog in red.

**Figure S10. The distribution difference of NS.** For each species, the left and right columns indicate nonessential and essential genes respectively. The blue, green, yellow and red bars indicates the frequency of the genes with 0~100, 100~200, 200~300 and >300 genomes that have orthologous hits, respectively.

**Figure S11. The distribution difference of NEH.** For each species, the left and right columns indicate nonessential and essential genes respectively. The blue, green, red bars indicates the frequency of the genes with 0, 1~8, and >8 essential homologous genes in remain 20 species.

**Figure S12. The distribution difference of NNH.** For each species, the left and right columns indicate nonessential and essential genes respectively. The blue, green, red bars indicates the frequency of the genes with 0, 1~8, and >8 non-essential homologous genes in remain 20 species.

**Figure S13. The distribution difference of mE, mEF, and Age in *SCE* and *SPO*.** The blue and red histograms indicate nonessential and essential genes respectively. The Age 5, 4, 3, 2, 1 from left to right are corresponding to cellular organisms, Eukaryota, Opisthokonta, Ascomycota, and species typical, respectively.
