# Supplementary material for: A new computational strategy for predicting essential genes
Source: BMC Genomics. 2013 Dec 21;14:910. doi: 10.1186/1471-2164-14-910 (PMC3880044; doi:10.1186/1471-2164-14-910)
Supplement: Additional file 6 — Calculation of the score vector S i for non-continuous features. [file 1471-2164-14-910-S6.doc]

**Supplemental Method**

**Calculation of the score vector $S_{i}$ for non-continuous features**

The non-continuous features may be divided into ordinal type and nominal type. The former can be treated as ordinal when its values represent categories with some intrinsic ranking (for example, the gene Age we defined). The latter can be treated as nominal when its values represent categories with no intrinsic ranking (e.g., domain type).

For these two types, we supposed the probability of one gene with a value or category (*x*) was *θ* and the number of genes (*k*) with the value or category (*x*) followed a binomial distribution *B(m,θ)*. To estimate the parameter *θ*, we employed bayes estimation strategy and supposed *θ* had a prior distribution $\pi(\theta)=1,\theta\in(0,1)$. Then, we used mean posterior estimate to obtain the MSE of *θ*:

$\hat{\theta}=E(\theta|k)=(k+1)/(m+2)$.

Based on the estimation of *θ*, we can obtain the probability of essential and non-essential gene with a value (*x_ij_*):

$p(x_{ij}|E)=(n_{jE}+1)/(N_{E}+2),p(x{}_{ij}|\bar{E})=(n_{j\bar{E}}+1)/(N_{\bar{E}}+2)$.

We then get the score vector , where $n_{jE}$ and $n_{j\bar{E}}$ represent the number of essential and non-essential genes having the value $x_{ij}$, respectively, and $N_{E}$ and $N_{\bar{E}}$ represent the total number of essential genes and that of non-essential genes in the genome.

**Reference**

1. Lehmann EL, Casella G: **Theory of point estimation**, vol. 31: Springer; 1998.

2. Berger JO: **Statistical decision theory and Bayesian analysis**: Springer; 1985.
